# A gene expression resource generated by genome-wide *lacZ* profiling in the mouse

**DOI:** 10.1242/dmm.021238

**Published:** 2015-11-01

**Authors:** Elizabeth Tuck, Jeanne Estabel, Anika Oellrich, Anna Karin Maguire, Hibret A. Adissu, Luke Souter, Emma Siragher, Charlotte Lillistone, Angela L. Green, Hannah Wardle-Jones, Damian M. Carragher, Natasha A. Karp, Damian Smedley, Niels C. Adams, Sanger Institute Mouse Genetics Project, James N. Bussell, David J. Adams, Ramiro Ramírez-Solis, Karen P. Steel, Antonella Galli, Jacqueline K. White

**Affiliations:** 1 Wellcome Trust Sanger Institute, Hinxton, Cambridge, CB10 1SA, UK; 2 Centre for Modeling Human Disease, Toronto Centre for Phenogenomics, 25 Orde Street, Toronto, CanadaM5T 3H7

**Keywords:** Gene expression, *lacZ* reporter, Mouse, Resource

## Abstract

Knowledge of the expression profile of a gene is a critical piece of information required to build an understanding of the normal and essential functions of that gene and any role it may play in the development or progression of disease. High-throughput, large-scale efforts are on-going internationally to characterise reporter-tagged knockout mouse lines. As part of that effort, we report an open access adult mouse expression resource, in which the expression profile of 424 genes has been assessed in up to 47 different organs, tissues and sub-structures using a *lacZ* reporter gene. Many specific and informative expression patterns were noted. Expression was most commonly observed in the testis and brain and was most restricted in white adipose tissue and mammary gland. Over half of the assessed genes presented with an absent or localised expression pattern (categorised as 0-10 positive structures). A link between complexity of expression profile and viability of homozygous null animals was observed; inactivation of genes expressed in ≥21 structures was more likely to result in reduced viability by postnatal day 14 compared with more restricted expression profiles. For validation purposes, this mouse expression resource was compared with Bgee, a federated composite of RNA-based expression data sets. Strong agreement was observed, indicating a high degree of specificity in our data. Furthermore, there were 1207 observations of expression of a particular gene in an anatomical structure where Bgee had no data, indicating a large amount of novelty in our data set. Examples of expression data corroborating and extending genotype-phenotype associations and supporting disease gene candidacy are presented to demonstrate the potential of this powerful resource.

## Abstract (precis)

**Editor's Choice:** The report presents an open-access adult mouse expression resource, in which the expression profile of 424 genes has been assessed in up to 47 different organs, tissues and sub-structures using a *lacZ* reporter gene.

## INTRODUCTION

The availability of robustly annotated genome sequences has enabled the follow-on challenge of assigning functional annotations to each gene. However, our ability to predict gene function from DNA sequence is poor. Compounding the challenge is a trend to focus activities on a relatively small proportion of previously characterised genes, leading many others to be ignored, the function of which remains unknown (Edwards et al., 2011; Pandey et al., 2014).

Pivotal to understanding the function of a gene is knowledge of its temporal and spatial expression pattern. There are many protein- and RNA-based approaches to define gene expression. The bulk of available adult gene expression data is derived from RNA-based technologies. Projects using these approaches include GenePaint (www.genepaint.org) and the Allen Brain Atlas (www.brain-map.org), both of which focus on the brain. Most of the data from these expression studies have been reported in federated databases presenting a composite of many distinct data sources, such as BioGPS (www.biogps.org) and Bgee (http://bgee.unil.ch/). RNA *in situ* hybridisation methodologies typically provide tissue sub-structure or cellular resolution on a small number of organs and tissues, whilst analyses of the transcriptome, using technologies such as RNA-Seq and microarray, often encompass a broader range of organs and tissues but do not offer resolution beyond the whole-organ level.

The International Mouse Phenotyping Consortium (IMPC) is a co-ordinated effort to generate and characterise a knockout mouse for 20,000 protein-coding genes (Brown and Moore, 2012; www.mousephenotype.org). The Wellcome Trust Sanger Institute is the single largest contributor to this effort, having generated more than 1500 knockout mouse lines and substantively characterised more than 1000 of these as of July 2015 (White et al., 2013; www.mousephenotype.org). The *lacZ* reporter gene is an integral part of the targeted allele design used by the IMPC. *lacZ* encodes bacterial β-galactosidase, which is a well-characterized enzyme for which methods of *in situ* localisation are widely available for embryo and adult tissues from multiple species. The protocol generally uses a simple and robust histochemical approach involving the substrate X-Gal (5-bromo-4-chloro-3-indolyl-β-d-galactopyranoside; Lojda, 1970). When X-Gal is oxidized by β-galactosidase in the presence of iron-containing compounds, a non-diffusible, insoluble, blue precipitate is produced, which can be visualised and imaged readily. This protocol is rapid and easy to implement, with a relatively good penetration in tissues and a high specificity. These attributes make this method, combined with other phenotypic tests, a powerful standard procedure to evaluate new genes of interest in a high-throughput environment.

**RESOURCE IMPACT**

**Background**

Systematic characterisation of mammalian gene function is a topical goal that forms the basis of an ambitious and on-going international effort termed the International Mouse Phenotyping Consortium (IMPC). Knowledge of the pattern of expression of a gene is a cornerstone piece of information in the quest to understand its normal function and any potential roles in disease. Definition of the expression profile of a gene can be achieved using different methods and techniques that are either RNA or protein based. One well-established technique makes use of a *lacZ* reporter gene that is integral to the targeted allele design at the centre of the IMPC. This reporter gene is expressed under the control of the endogenous regulatory elements of the gene of interest. Whilst the methodology to detect expression of the reporter gene is well established, the scale and ambition of the project is unprecedented.

**Results**

In this study, the investigators determined the expression profile of 424 genes using whole-mount techniques to detect expression of a *lacZ* reporter. This represents the largest gene set for which an adult mouse expression profile has been published using this approach. Up to 47 tissues were assessed for each gene. All the data are available as an open access resource (www.mousephenotype.org/). The investigators noted many new, specific and informative expression patterns. Expression was most commonly observed in the testis and brain and was most restricted in white adipose tissue and mammary gland. When grouped by the number of positive structures, over half of the assessed genes presented with an absent or localised expression pattern (0-10 tissues). Genes that were widely expressed (≥21 tissues) were more likely to present with reduced postnatal viability of homozygous progeny. The investigators presented examples of expression data corroborating, extending and informing genotype-phenotype associations and supporting disease candidacy.

**Implications and future directions**

The IMPC aims to perform systematic characterisation of 20,000 protein-coding genes. The authors presented expression data for more than 2% of that target. This large contribution has resulted in optimisation and streamlining of the procedure to support the accelerated throughput required to deliver the IMPC goal in a timely manner. This powerful and diverse, open access gene expression resource will extend the scientific community's understanding of normal organ function and physiology as well as of the origins and progression of many diseases.

We present to the scientific community an open access adult expression resource, in which the profile of 424 genes was determined using high-throughput, *lacZ* reporter gene whole-mount analysis. Expression in up to 47 organs, tissues and sub-structures (herein referred to as tissues) was assessed for each gene. This represents the single largest gene set for which an adult mouse expression profile has been published using the *lacZ* reporter gene.

## RESULTS

### Genes and alleles

Mice carrying targeted gene knockout alleles from the EUCOMM and KOMP embryonic stem cell resources (Fig. S1; Skarnes et al., 2011) were generated on a C57BL/6 genetic background. One of the following allelic configurations was studied for each of the 424 genes included in this study: either the knock-in allele typically referred to as ‘tm1aWtsi’ or the deletion allele (referred to as ‘tm1bWtsi’), which is derived from the tm1aWtsi allele by *Cre*/*loxP*-mediated excision of the critical exon (Fig. S1). Integral to the tm1aWtsi and tm1bWtsi allele design was a *lacZ* reporter gene. The mutants generated are listed in Table S1 and are available through public repositories, including the European Mutant Mouse Archive (http://www.emmanet.org/) and KOMP (https://www.komp.org/). For 25 genes assessed initially as tm1bWtsi, comparison with the parental tm1aWtsi allele was performed retrospectively, as detailed below.

### Characterisation of mice

**Fig. 1.**
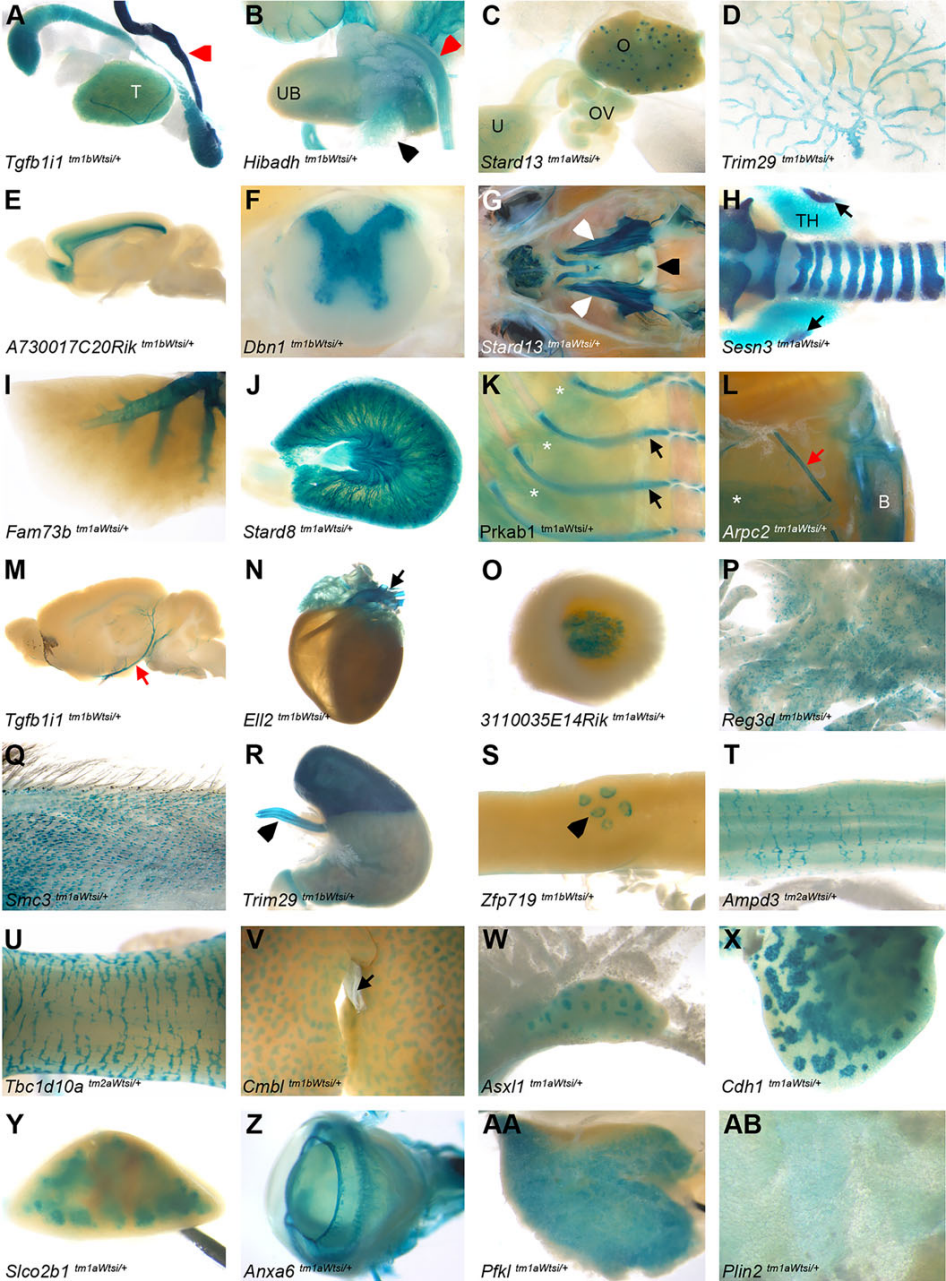
**The endogenous expression pattern of each gene was assessed in up to 47 tissues.** *lacZ* reporter gene whole-mount analysis was performed to ascertain the expression profile of each gene of interest. Examples are presented of expression in each of the entities annotated. The genotype is stated for each panel. (A) Testis (T); epididymis was excluded because of endogenous background staining. (B) Male urogenital system with prostate (black arrowhead) and urinary tract staining on the urinary bladder (UB). (A,B) Vas deferens (red arrowhead). (C) Female reproductive system with the ovary (O), oviduct (OV) and uterus (U). (D) Mammary gland. (E) Brain (inner cerebral cortex; see Fig. 3 for expression in other annotated brain sub-structures). (F) Spinal cord grey matter. (G) Cranial cavity including the pituitary (black arrowhead) and peripheral nerve (trigeminal ganglia) staining (white arrowheads). (H) Trachea, thyroid (TH) and parathyroid glands (arrows). (I) Lung. (J) Kidney. (K) Cartilaginous rib (black arrows). (K,L) Skeletal muscle (asterisks). (L) Hindlimb with bone staining (B) on the tibia. (L,M) Vascular staining on blood vessels (red arrow). (N) Heart and aorta (arrow). (O) Adrenal medulla. (P) Pancreas. (Q) Skin. (R) Stomach and oesophagus (arrowhead). (S) Small intestine and Peyer's patches (arrowhead). (T) Large intestine. (U) Colon. (V) Liver and gall bladder (arrow). (W) Mesenteric lymph nodes. (X) Thymus. (Y) Spleen. (Z) Eye. (AA) Brown adipose tissue. (AB) White adipose tissue.

Mice for each mutant allele were characterised using a standardised battery of clinical phenotyping procedures, as described previously (White et al., 2013) and summarised in Fig. S2. In addition to this phenotypic characterisation, the endogenous expression pattern of each targeted gene was assessed in adult mice by taking advantage of the inserted *lacZ* reporter gene (Figs S1 and S2A). Animals heterozygous for the targeted allele were typically used for whole-mount expression analysis in order to reduce the possible confounding effects of the loss of gene function. The whole-mount *lacZ* reporter gene workflow presented in Fig. S2A was used to assess expression in tissues, as highlighted in Fig. 1 (see also Table S1). Data and images from the phenotypic and expression characterisation can be viewed on the International Mouse Phenotyping Consortium's website (http://www.mousephenotype.org), an integrated database searchable by gene, anatomical term, procedure, phenotype and disease association.

### Expression profile for each targeted gene

We present a snapshot of expression at a single time point for 424 unique genes (Table S1). Of these genes, 295 were reporter tagged via the knock-in allele (e.g. tm1aWtsi), whereas the remaining 129 were tagged via the deletion allele (e.g. tm1bWtsi). These genes are distributed on all chromosomes except Y (Fig. S3). Comparison of this gene set with all Gene Ontology (GO) annotated mouse genes revealed that it can be considered representative of the entire mouse genome (data not shown).

Expression was typically assessed in two mice (one male and one female; aged between 6 and 30 weeks) per targeted gene (Table S1). Of the tissues assessed for expression, six were sex-specific reproductive organs (ovaries, oviduct, uterus, testis, prostate and vas deferens). Epididymis was excluded because of endogenous background staining (Fig. S4I). The following four outcomes were used to describe each entity that was assessed. (1) ‘Present’ indicated that reporter gene expression was observed in that tissue. (2) ‘Not detected’ indicated that reporter gene expression was not observed. Given the snapshot nature of the screen, this outcome is not synonymous with the absence of expression, and may reflect that the target gene was expressed at levels below the detectable threshold of the assay, was not expressed in the context of the age and conditions of the experiment or was never expressed in the tissue being assessed. (3) ‘No data’ indicated that, for technical reasons, the data were missing. (4) ‘Ambiguous’ indicated that we were unable to make a confident assessment either because the reporter gene expression was so faint that it was barely perceptible or because of interference from endogenous background staining. The incidence, location and range of intensity of endogenous background was extensively assessed in wild-type mice (*n*=116). Glandular stomach, kidney and thyroid were the principal tissues that gave characteristic patterns of background staining (Fig. S4). Background was also detected to a lesser extent in bone, mesenteric lymph node, thymus, ovary and testis.

The data collected on individual mice were used to derive summary expression profiles for each gene, for up to 47 tissues assessed (Table S1) using the strategy outlined in the Materials and Methods section.

**Fig. 2.**
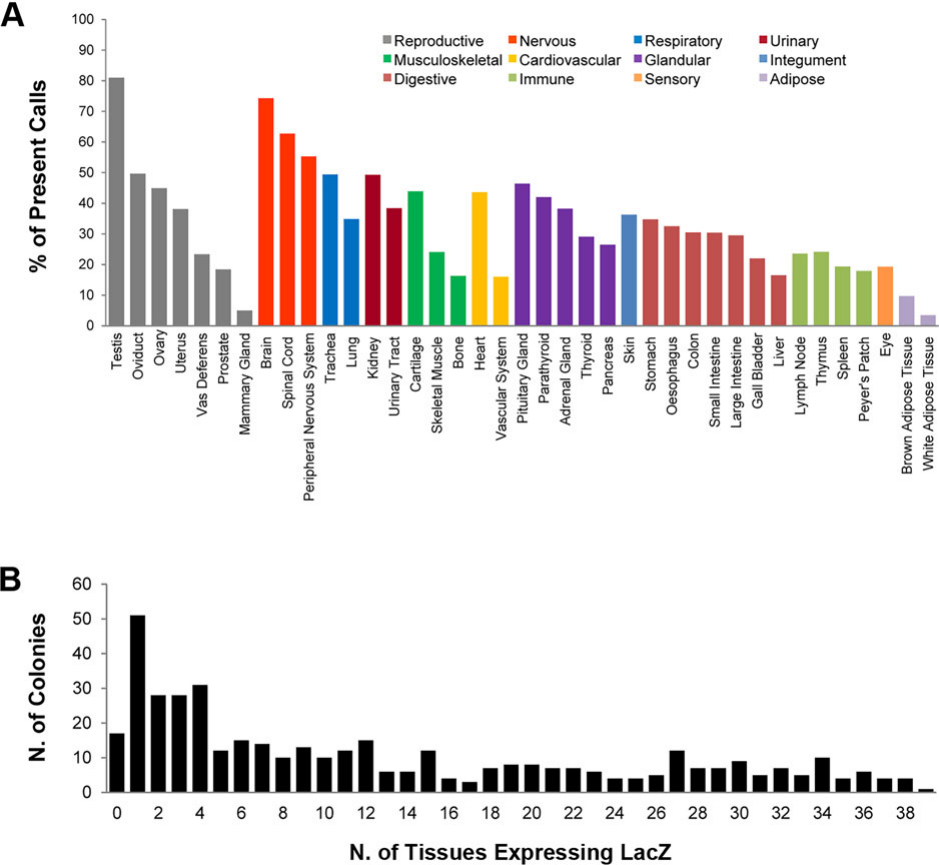
**Distribution and extent of expression.** (A) The relative percentage of genes for which *lacZ* reporter gene expression was detected for each of the 39 standard tissues, grouped by system. The percentage was calculated based on 424 genes, excluding instances of no data (Table S1). (B) For each gene, the total number of *lacZ* reporter-positive tissues is presented. The most common profile was expression in a single tissue (51 lines), and over half of the lines assessed (54.0%) presented with an absent or localised expression pattern (0-10 tissues).

For each of the 424 genes, a standard set of 39 tissues was assessed (16,536 possible outcomes). This analysis revealed 5404 calls of expression ‘present’ (32.7%), 10,295 ‘not detected’ (62.2%), 488 ‘ambiguous’ (3.0%) and 349 (2.1%) with ‘no data’ available for technical reasons. Table S2 breaks down these outcomes into categories based on the confidence of each call. The majority of ‘present’ and ‘not detected’ calls (12,115, 73.3% of all data) were derived from multiple mice and were fully concordant within our data set. Many specific and informative expression patterns were noted. Target gene expression was most commonly observed in the testis and brain, with 81.0 and 74.3% of genes assessed as ‘present’, respectively. Spinal cord and peripheral nervous system were the next most commonly expressed, ‘present’ in more than 55% of all genes included in this study. Expression was least frequently observed in adipose tissue (3.5%, 15/424 genes for white and 9.7%, 41/424 genes for brown) and mammary gland (5.0%, 21/418), although this may in part be a consequence of stain penetration (West et al., 2015). The percentage of expressed genes across all 39 structures assessed is presented in Fig. 2A. Interestingly, the nervous system is highly represented in that three-quarters of the screened genes are positive in brain, spinal cord and/or peripheral nervous system.

When grouped by the number of positive tissues (Fig. 2B), 17 lines (4.0%) showed complete absence of expression, 107 lines (25.2%) presented with limited tissue expression (1-3 positive structures), 105 lines (24.8%) were classed as localised expression (4-10 positive structures), whereas 195 (46.0%) were broadly expressed (≥11 structures). Interestingly, the most common profile was expression in one single tissue (51 lines), and over half of the lines assessed (54.0%) presented with an absent or localised expression pattern (0-10 tissues).

### Extending and improving the expression profile for key organs

X-Gal stain penetration was limited in dense and encapsulated organs, such as the brain, kidney and spleen. This caused some inconsistencies in staining between mice. To address this, thick midline sections of the brain (longitudinal) and kidney (transverse) were generated for staining, and a transverse bisection of the spleen performed. A comparison of the brain staining is presented in Fig. S5. This refinement was introduced for the final 125 unique genes processed and allowed annotation of seven additional sub-structures of the brain (brainstem, cerebellum, hypothalamus, hippocampus, striatum, cerebral cortex and olfactory lobe) in these lines (Table S1). Annotation of the aorta (Fig. 1N) was also included for this subset of lines, bringing the maximal number of structures annotated to 47.

**Fig. 3.**
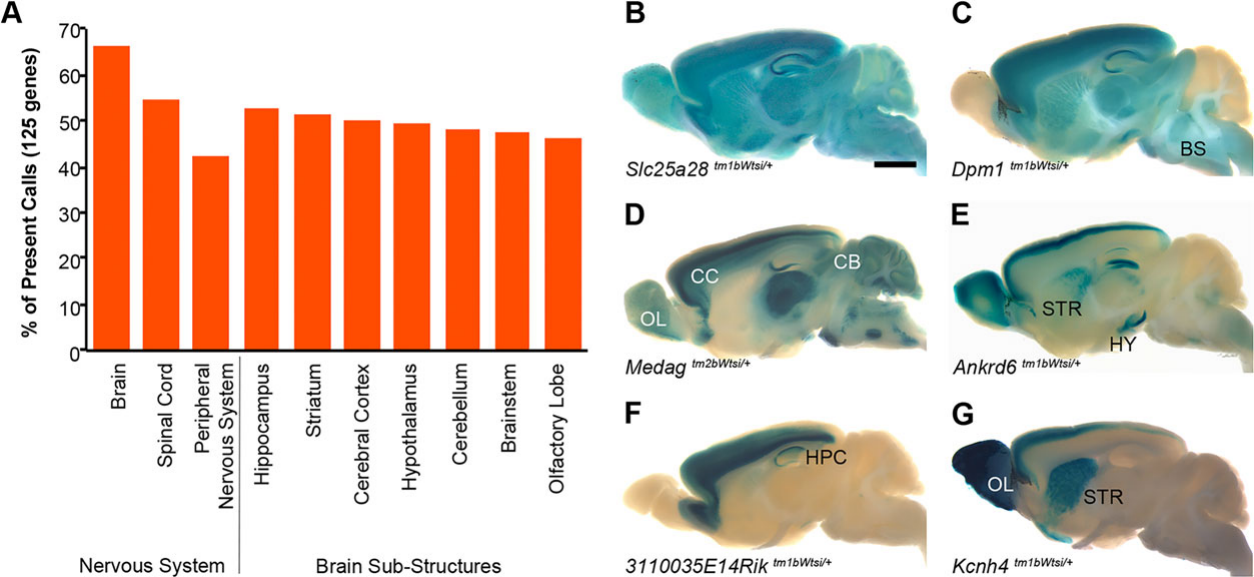
**The granularity of annotation was extended in the brain.** (A) The percentage of genes for which *lacZ* reporter gene expression was detected in the brain, spinal cord, peripheral nervous system and seven selected brain sub-structures. The percentage was calculated based on the subset of 125 genes for which the extended brain annotation was performed (Table S1). (B-G) Examples of expression patterns in the brain included the following: (B) ubiquitous expression of *Slc25a28*; (C,E) olfactory lobe, cerebral cortex, hippocampus, striatum, hypothalamus, cerebellum and brainstem expression of *Dpm1* (C) and *Ankrd6* (E); (D) olfactory lobe, cerebral cortex, hippocampus, hypothalamus, cerebellum and brainstem expression of *Medag*; (F) cerebral cortex, hippocampus and brainstem expression of *3110035E14Rik*; and (G) olfactory lobe, cerebral cortex and striatum expression of *Kcnh4*. Olfactory lobe (OL), cerebral cortex (CC), hippocampus (HPC), striatum (STR), hypothalamus (HY), cerebellum (CB) and brainstem (BS). Scale bar: 2 mm.

Expression was observed in the brain of 83 of the 125 genes for which this extended analysis was performed. For 53% (44/83) of this subset of genes with brain staining, all seven of the sub-structures were positive, indicating broad expression in the central nervous system for these genes (Fig. 3A). Examples of the patterns observed and the distribution of staining across each of the sub-structures are presented in Fig. 3B-G.

### Distinctive organ sub-distribution adds value

**Fig. 4.**
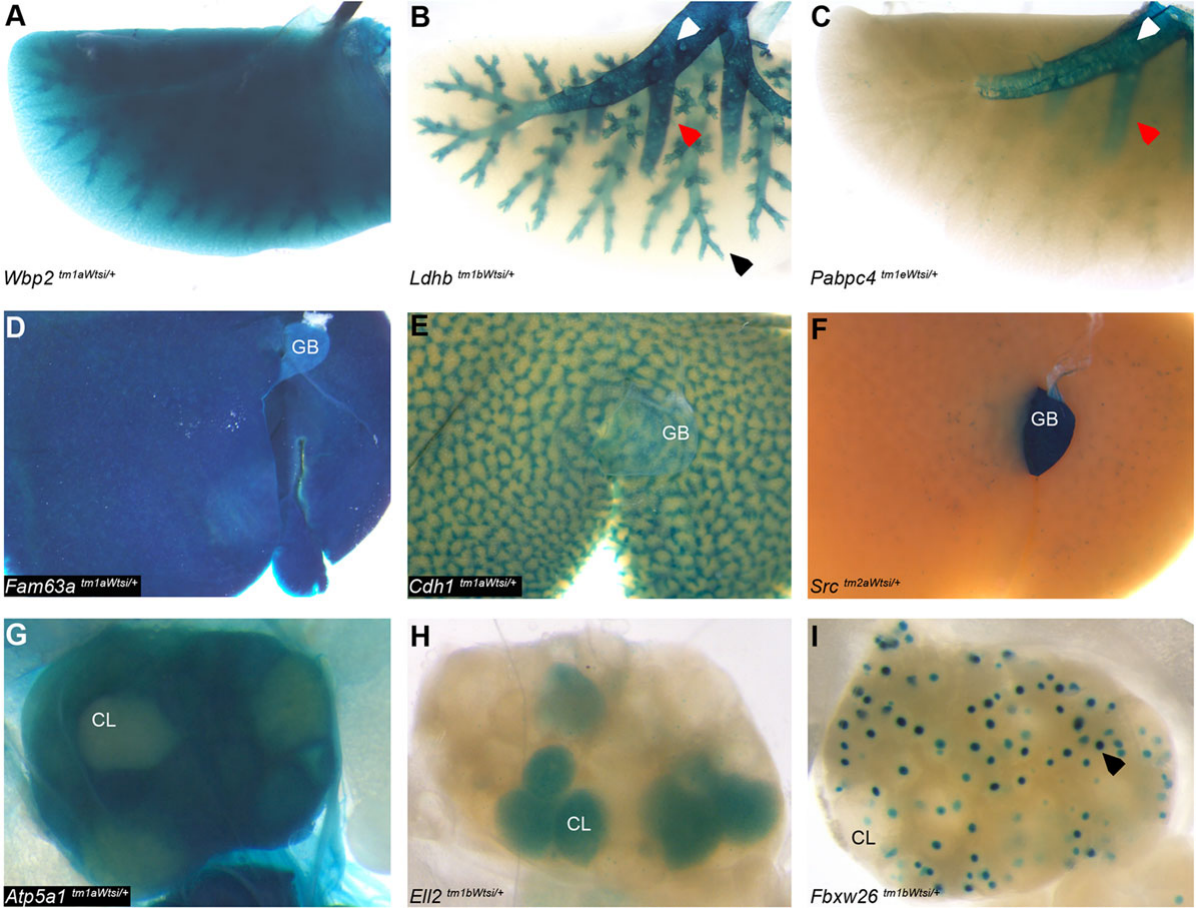
**Demonstration of additional granularity within the image resource.** The pattern of expression within each structure was not routinely annotated. Examples are presented of different classes of expression pattern found in lung, liver and ovary. The genotype is stated for each panel. In the lung examples (A-C), primary bronchi (white arrowhead), pulmonary bronchi (red arrowhead) and terminal bronchiole (black arrowhead) sub-structures are indicated. In the liver (D-F), the gall bladder is labelled (GB). (D) Diffuse expression throughout the liver. (E) Expression in portal areas delineates the classic portal lobules. (F) Specific expression in the GB, with punctate expression in the liver parenchyma that we speculate corresponds to biliary structures/ductules. In the ovary (G,H,I), the corpora lutea are labelled (CL). (G) Diffuse expression excluding the CL. (H) Selective expression in CL. (I) Selective expression in the ova within the ovarian follicles (black arrow).

To standardise data collection, we annotated each allele with a defined set of anatomical structures. This strategy allowed comparison between genes and resulted in minimal missing data. However, additional granularity can be extracted from the images via the patterns of expression within each structure, and many specific and informative expression patterns were noted. Examples are presented for structures annotated as lung, liver and ovary (Fig. 4). In the lung examples, the global expression for *Wbp2* is likely to represent expression in the entire lung parenchyma, including airways and alveoli (Fig. 4A). The branching pattern seen for *Ldhb* demonstrates expression in the primary bronchi, pulmonary bronchi and terminal bronchioles, with expression not detected in alveolar ducts, alveoli and blood vessels for this gene (Fig. 4B). *Pabpc4* presents with expression localised specifically to the primary and intrapulmonary bronchi (Fig. 4C). In the liver examples, the ubiquitous expression of *Fam63a* is likely to represent all hepatocytes, bile ductules and vasculature (Fig. 4D). The clear hexagonal outline of the hepatic lobules seen for *Cdh1*, epithelial cadherin, is indicative of hepatic arterioles or portal venules (Fig. 4E). The faint, punctate stain seen as a subtle hexagonal pattern in the liver parenchyma combined with the strong expression in the gall bladder (Fig. 4F) leads us to postulate that *Src* liver staining may represent biliary ductules. *Atp5a1* showed diffuse staining in all compartments of the ovary except the corpora lutea (Fig. 4G), whereas, within the ovary, *Ell2* is detected only in the corpora lutea (Fig. 4H). By contrast, *Fbxw26* was expressed in the ova within the ovarian follicles (Fig. 4I). Expression of genes within the ovary may be influenced by the stage of oestrus at the time of collection, and this should be considered.

### Comparison with published expression data

For validation purposes, our data from all 424 genes and 47 tissues were compared with the Bgee gene expression database (http://bgee.unil.ch/bgee/bgee; Bastian et al., 2008), which includes RNA-Seq, microarray, *in situ* hybridisation and expressed sequence tag data. Out of 424 genes, a subset of 339 had one or more call of ‘present’ in our data set and expression data on Bgee. Comparing the 100% consistent in-house ‘present’ calls with Bgee showed that there was a high degree of agreement between the data resources (Fig. S6). These results suggest that the specificity of the in-house data was high. There were 1207 100% consistent in-house calls of ‘present’ for structure-gene combinations for which Bgee had no data, indicating that this publication presents a large amount of new data. Structures that the in-house data were particularly enriched for included peripheral nervous system, cartilage, trachea, urinary tract, thyroid and parathyroid glands (data not shown).

### Influence of allele design on expression pattern

Of the 424 unique genes characterised, 309 retained the neomycin (*neo*) selectable marker, of which 132 were configured to be expressed via the *β-actin* or *phosphoglycerate kinase 1* promoter, whilst 177 carried a promoterless *neo* selectable marker (Fig. S1). Analysis did not reveal any expression patterns indicative of consistent interference from the presence of either configuration of *neo*. However, the following pattern did emerge relating to the complexity of the expression profile. Genes targeted with the promoterless construct were more likely to have a larger number of positive structures (Table S1; median number ‘present’=20, mean=19.1, minimum=0, maximum=39) than genes targeted with the promoter-driven construct (median number ‘present’=4, mean=7.9, minimum=0, maximum=34; *P*<0.0001, Mann-Whitney *U*-test).

For 25 genes assessed initially as tm1bWtsi, comparison with the parental tm1aWtsi allele (Fig. S1) was performed retrospectively, using Bgee data as a reference. A modest pattern emerged. Statistically, tm1bWtsi gave more calls of ‘present’ that were consistent with Bgee data compared with tm1aWtsi (*P*=0.037, Wilcoxon signed-rank test with continuity correction). This equated to one additional ‘present’ expression call when looking at the median difference between alleles. Whilst statistically significant, the effect size was small, as was the sample size. More analysis would be required to confirm and investigate this difference further, but this was beyond the scope of this study.

### Expression profile complexity is correlated with homozygous viability

**Fig. 5.**
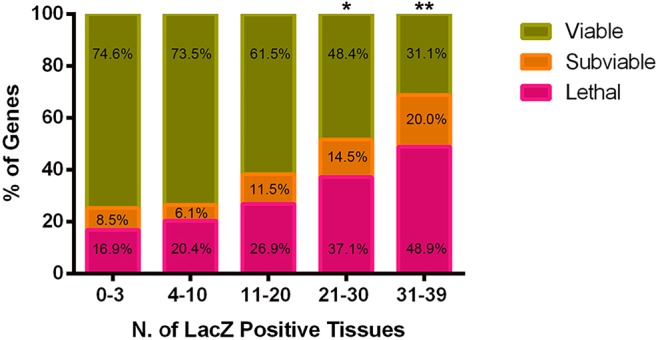
**The complexity of expression profile relates to homozygous viability.** For each gene, the total number of *lacZ* reporter-positive tissues was calculated and grouped into one of five categories (0-3, 4-10, 11-20, 21-30 and 31-39 positive structures). Within each category, the percentage of genes for which homozygous mice were lethal, subviable and viable at postnatal day 14 (Table S3) is presented. Fisher's exact test was used to compare the ratio of outcomes in the 0-3 group with each of the other groups (*P<0.001; **P<0.0001).

Assessing the data as a whole highlighted a trend linking the complexity of the expression profile to the viability of homozygous/hemizygous mice. Typically, viability was assessed at postnatal day 14 (P14) by genotyping live progeny from heterozygous crosses. This was completed for 401 of the 424 genes (Table S1). Of these 401 genes, 62.9% (252/401) were classed as viable, whereas 26.4% (106/401) were classed as lethal because they produced no homozygous mice at P14. The remaining 10.7% (43/401) produced less than half of the expected number of homozygous progeny and were therefore classed as subviable. The distribution of P14 viability calls was screened against the number of tissues expressing the *lacZ* reporter gene. These data were grouped into genes with absent or limited tissue expression (0-3 positive structures), localised expression (4-10 positive structures) and three additional sub-classes that represented increasing complexity of expression (11-20, 21-30 and 31-39 positive structures; Fig. 5 and Table S3). Overall, genes expressed in a restricted number of tissues were more likely to be viable at P14 compared with genes expressed in a more generalised manner. Indeed, genes expressed in 31-39 tissues were most likely to be lethal at P14 [*P*<0.0001, Fisher's exact test comparing the ratio of viable and abnormal viability (subviable and lethal combined) gene sets between the groups with 0-3 and 31-39 positive structures]. Comparison of the viable and abnormal viability gene sets with all GO annotated mouse genes revealed an increased complexity of function in the abnormal viability genes, as this group possessed 0.7% (125/16,953) over-represented GO terms. In comparison, the viable gene group showed 0.04% (8/16,953) over-represented GO terms (data not shown). Both groups showed a low level of under-represented GO terms.

### Expression data confirm and extend existing knowledge

**Fig. 6.**
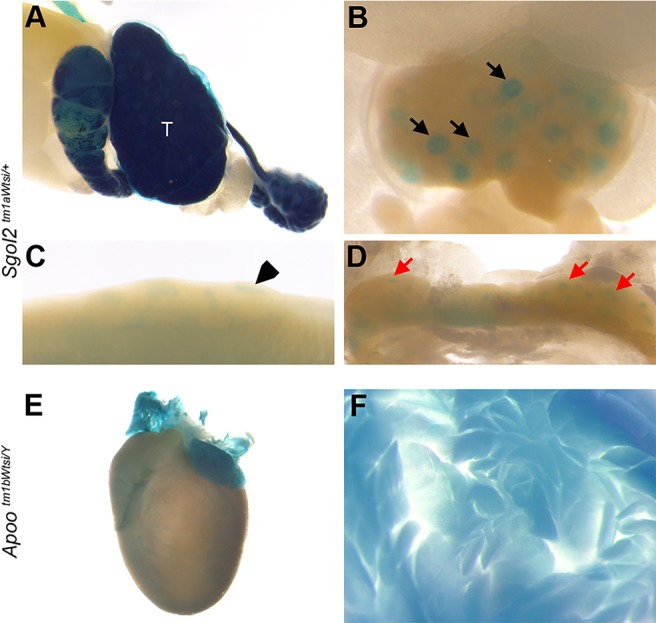
**Expression data confirm and extend existing knowledge.** *lacZ* reporter gene expression in *Sgol2^tm1aWtsi/+^* mice in the following tissues: (A) testis (T); (B) ovary (black arrows); (C) Peyer's patches (black arrowhead); and (D) mesenteric lymph node (red arrows). (E,F) Expression in *Apoo^tm1bWtsi/Y^* mice in heart (E) and pancreas (F).

In addition to the new information provided by those gene expression patterns that have not been reported in adults previously, there is also significant potential to confirm and extend our knowledge of those genes with existing expression data, because the methodology used provides high resolution for a wide range of tissues. One such example is *Sgol2*, which encodes the cohesion protein shugoshin-like 2. BioGPS (http://biogps.org/#goto=genereport&id=68549) reports strong *Sgol2* expression in cell lines and tissues with actively dividing cells, such as the testis and bone marrow, supporting a function for shugoshin-like 2 to protect centromeric cohesion. In addition, low *Sgol2* expression levels were associated with meiosis segregation defects in aged eggs (Yun et al., 2014). The function of shugoshin-like 2  is supported by Llano et al. (2008), who reported *Sgol2* expression in the testis. We confirmed and extended these observations, reporting *lacZ* expression in the testis, ovary, Peyer's patches, mesenteric lymph nodes (Fig. 6A-D), thymus and spleen (data not shown) of *Sgol2^tm1aWtsi/+^* mice; all of these tissues contain actively dividing cells.

*Apoo*, which encodes apolipoprotein O, has recently been implicated in diabetes-related heart disease (Turkieh et al., 2014). This mitochondrial protein is thought to play a role in regulating lipid accumulation in the heart of obese and diabetic individuals. We detected *lacZ* expression in the heart and pancreas of *Apoo^tm1aWtsi/Y^* and *Apoo^tm1aWtsi/+^* mice (Fig. 6E,F).

### Expression data corroborate and inform genotype-phenotype associations

**Fig. 7.**
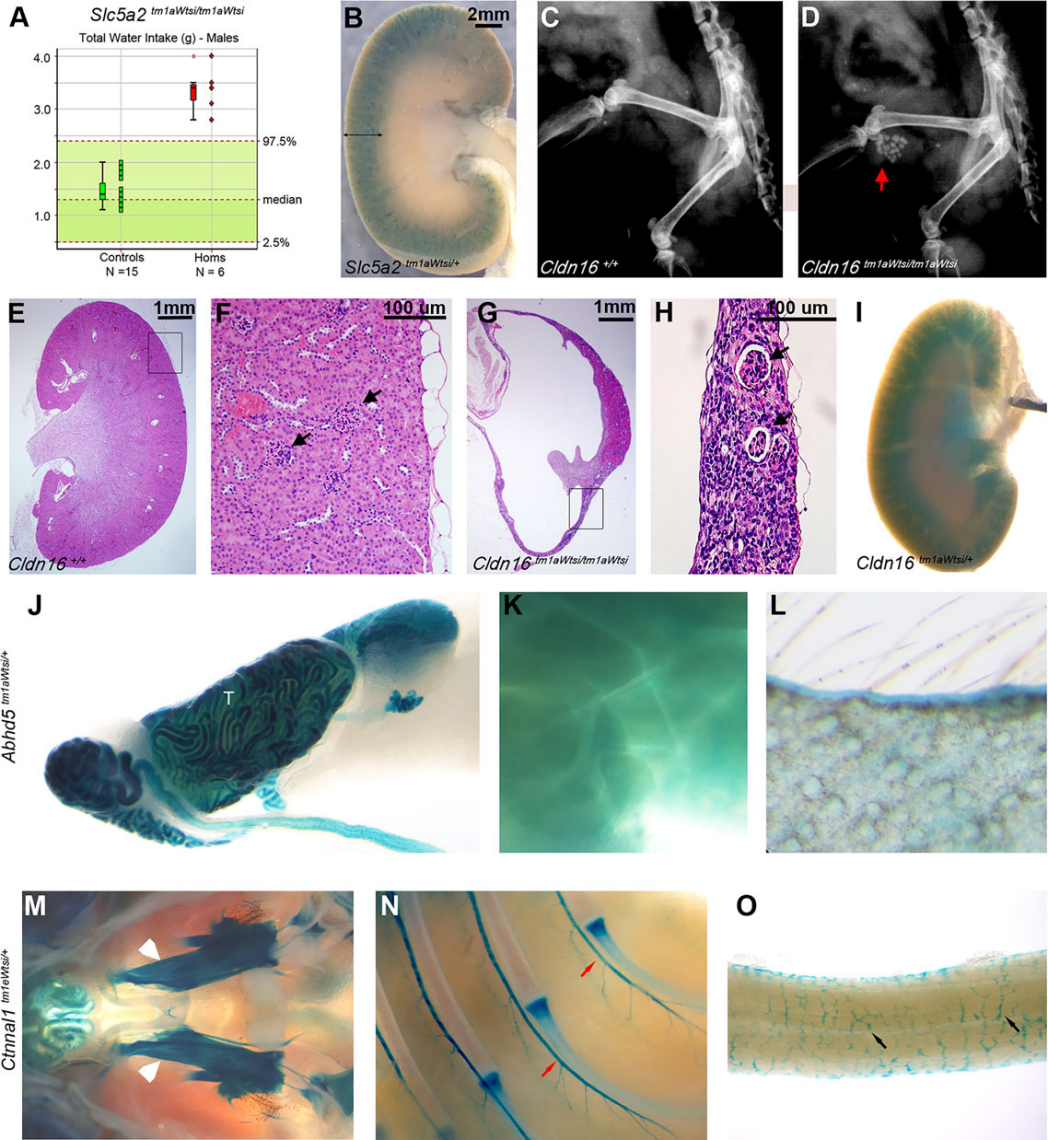
**Expression data inform genotype-phenotype associations.** (A) Increased water intake of *Slc5a2^tm1aWTsi/tm1aWTsi^* males (*n*=6; red symbols) over a 21 h interval compared with local controls (*n*=15; green symbols) and the 95% reference range (*n*=656). Each mouse is represented as a single symbol on the graph. Median, 25th and 75th percentiles (box) and the lowest and highest data point still within 1.5× the interquartile range (whiskers) are shown. (B) *lacZ* reporter gene expression in *Sgol2^tm1aWtsi/+^* mice in kidney cortex (scale bar: 2 mm). (C,D) Urolithiasis (red arrow) shown by X-ray in all *Cldn16^tm1aWtsi/tm1aWtsi^* males (*n*=7; D) compared with wild-type controls (C). (G,H) Histopathology showed hydronephrosis in both sexes [glomeruli, black arrows; scale bar: 1 mm (G) and 100 μm (H)] compared with wild type (E,F). *lacZ* reporter gene expression in the following tissues: (I) kidney cortex of *Cldn16^tm1aWtsi/+^* mice; testis (T; J), brown adipose tissue (K) and skin (L) of *Abhd5^tm1aWtsi/+^* mice; and trigeminal ganglia (white arrowheads; M), intercostal nerves (red arrows; N) and enteric nervous system (black arrows; O) of the large intestine of *Ctnnal1^tm1eWtsi/+^* mice.

*SLC5A2* encodes the sodium-dependent glucose transport protein solute carrier family 5, member 2, dysfunction of which is associated with renal glucosuria. In addition to renal glucose wasting, affected individuals present clinically with polyuria and polydipsia (Santer and Calado, 2010). Mice homozygous for a *Slc5a2* null allele were reported previously (Vallon et al., 2011) to phenocopy many aspects of the human disease, including increased urine glucose and drinking behaviour. Using immunohistochemistry, Vallon et al. (2011) localised Slc5a2 expression to the brush border membrane of proximal convoluted tubules, located in the kidney cortex. We confirmed increased water intake in *Slc5a2^tm1aWtsi/tm1aWtsi^* male mice (Fig. 7A). Furthermore, *lacZ* expression was detected in the cortex of the kidney (Fig. 7B), as well as in mesenteric lymph node, ovary, uterus and testis (data not shown) of *Slc5a2^tm1aWtsi/+^* mice. This expression profile supports the published immunohistochemistry data and highlights two additional biological systems of potential clinical relevance in *SLC5A2*-deficient individuals.

CLDN16 dysfunction is associated with renal hypomagnesaemia 3. A mouse model of *Cldn16* deficiency resulted in serum hypomagnesaemia and hypercalciuria (Will et al., 2010); however, structural defects of the kidney and urinary bladder were not reported. We observed urolithiasis in the form of urinary bladder stones in *Cldn16^tm1aWtsi/tm1aWtsi^* male mice (Fig. 7C,D) and severe hydronephrosis in both sexes (Fig. 7E-H). Furthermore, we detected *lacZ* expression in eight tissues of *Cldn16^tm1aWtsi/+^* mice, including the kidney (Fig. 7I and Table S1).

Defects in ABHD5 are known to cause Chanarin-Dorfman syndrome, a rare form of non-bullous congenital ichthyosiform erythroderma (Angelini et al., 1980). This is a condition that primarily affects the skin and is caused by abnormal storage of neutral lipids. No skin abnormalities were detected in *Abhd5^tm1aWtsi/tm1aWtsi^* males. However, histopathology detected a testicular lesion characterized by marked vacuolation of the Sertoli cells suggestive of lipid storage (Adissu et al., 2014). Interestingly, expression analysis of *Abhd5^tm1aWtsi/+^* mice supported this testicular defect and demonstrated the extent to which *lacZ* was also expressed in the skin (pinna and tail), the major tissue affected in individuals with Chanarin-Dorfman syndrome, and in white and brown adipose tissues (Fig. 7J-L), where Abhd5 could play additional functional roles.

### Expression data support disease candidacy

Hirschsprung disease (HSCR) is a polygenic disorder affecting the enteric nervous system. Loss of innervation in the colon results in defective peristalsis, which causes chronic constipation and abdominal distension. *RET* is the primary HSCR gene, accounting for 50% of familial cases; however, many modifier loci have been proposed, including two associated regions mapping to chromosome 9q31 (Tang et al., 2010a). Region 9q31A represents a gene-rich location containing four genes, including *CTNNAL1* (Tang et al., 2010a). Microarray data previously indicated that *Ctnnal1* was a gene expressed in the enteric nervous system (Heanue and Pachnis, 2006). *Ctnnal1^tm1eWtsi/+^* expression analysis confirmed strong, broad staining in the brain and peripheral nervous system, including trigeminal ganglia, intercostal nerves and the enteric nervous system (Fig. 7M-O). This whole-mount expression analysis provides support to the candidacy of *Ctnnal1* as a modifier of HSCR.

## DISCUSSION

We present the expression profile of 424 mouse genes, determined by high-throughput, genome-wide whole-mount analysis using the β-galactosidase (*lacZ*) reporter gene. Qualitative assessment (Juers et al., 2012) of the expression of each gene in up to 47 tissues is available as an open access resource (www.mousephenotype.org/). This represents the largest gene set for which an adult mouse expression profile has been published using the *lacZ* reporter gene, and is part of an international effort to extend the functional annotation of the mammalian genome (Brown and Moore, 2012).

Defining the expression profile for a gene can be achieved using different methods and techniques. Our resource extends other protein-based expression data sets, including smaller-scale efforts using a similar methodology (Valenzuela et al., 2003; West et al., 2015) and protein-specific antibody studies (Helsby et al., 2014). Furthermore, it complements the many RNA-based adult and embryo expression data sets, such as the Allen Brain Atlas (Sunkin et al., 2013) and Eurexpress (Diez-Roux et al., 2011), as well as gene annotation portals presenting a federation of genetic and genomic resources, such as BioGPS (Wu et al., 2009) and Bgee (Bastian et al., 2008).

The philosophy adopted in this study regarding the assignment of expression status was conservative, as our focus was on building a high-quality resource. The concordance in our data was 83.3% if we include calls of ‘present’ derived from at least two biological replicates. This is similar to the equivalent measure of concordance reported in a recent methods comparison study involving *lacZ* whole-mount analysis (76.6%; West et al., 2015).

It is important to be cognisant of the limitations of the resource. The scale of the project necessitated that we capture a snapshot of expression in a small number of adult mice, typically two, up to a maximum of eight mice per gene. As with any expression analysis, the observed profile will be influenced by factors including age, sex, genetic background, environmental conditions (e.g. diet, temperature) and health status as well as cyclical modifiers, such as diurnal, oestrus and seasonal variation. Expression of liver (B-type) phosphofructokinase (*Pfkl*) illustrates this point. We detected *Pfkl* expression in 15 tissues (Table S1), but this did not include the liver. *Pfkl* expression in liver is highly influenced by the metabolic state of the animal, being upregulated in fasted mice (Gehnrich et al., 1988), which may explain our results. Our experimental design controlled many of these factors to reduce the variability within the data set, but some limitations remained. First, endogenous background can arise using *lacZ* whole-mount analysis, although the distribution and intensity vary within and between studies (Weiss et al., 1997; Adams and Gale, 2006; Odgren et al., 2006; Kopp et al., 2007; Bolon, 2008; West et al., 2015). The background is thought to arise from the presence of β-galactosidase activity in bacteria, e.g. in the gastrointestinal tract and at the surface of the teeth, or from cross-reactivity of other enzymes hydrolysing the X-Gal substrate. The latter source was controlled, but not eliminated, by closely regulating the fixative used, and the pH and temperature of the histochemical reaction (Weiss et al., 1999; Ma et al., 2002; Verma and Asakura, 2011; Shimada et al., 2012). This background staining was fully considered during manual annotation of the expression profile. Second, although the specificity of the results was high, the results were limited by sensitivity. For example, inactivation of cyclin-dependent kinase inhibitor 2A (*Cdkn2a*) has been reported previously to cause severe eye-related phenotypic consequences (McKeller et al., 2002). This was observed in-house (www.mousephenotype.org/data/genes/MGI:104738 and data not shown). *Cdkn2a* is reported to be expressed at low levels in the adult eye (http://biogps.org/#goto=genereport&id=12578); however, we detected *Cdkn2a* expression in the testis only (Table S1). This limitation in sensitivity was in part a consequence of the experimental design for a high-throughput, large-scale expression analysis screen where a delicate balance of throughput and sensitivity needed to be struck. It was further confounded by the technical limitation that whole-mount sensitivity is typically lower than that achieved using immunohistochemistry (Couffinhal et al., 1997; Pereira et al., 2006) or RNA hybridisation techniques. For this reason, lack of expression was recorded as ‘not detected’ and was not synonymous with ‘absent’. Third, the whole-mount technique limits the resolution to selected sub-structures; cell types are not differentiated. Our workflow was designed to balance throughput with depth of analysis and, as such, a maximum of 47 tissues were selected for annotation. We demonstrated additional granularity in the images from this resource (Fig. 4) and encourage the development of automated annotation tools that could realise the full potential of all enterprises of this type that are on-going in the community by delivering annotation of a broader list of entities. Histological evaluation would be required to investigate cellular localisation, and tissues suitable for histology are available upon request.

The selectable marker and nature of the allele have the potential to affect the expression pattern. Whilst corroboration of our findings using an alternative methodology, such as *in situ* hybridisation, was not performed, no consistent system-wide patterns were observed indicative of interference from the presence of either configuration of the neomycin selectable marker, promoter-driven or promoterless *neo*. Interestingly, a pattern relating to the complexity of expression profile was noted. Genes targeted with a promoterless *neo* cassette were more likely to display reporter gene expression in a large number of tissues. These genes must be expressed in embryonic stem cells in order for the targeting event to be successful. This observation may therefore reflect a difference in the typical expression profile of the class of genes that can be targeted using a promoterless strategy. We performed a small comparative study (*n*=25 genes) to investigate the difference in expression profile obtained from a tm1aWtsi or tm1bWtsi targeted allele (Fig. S1). Tm1bWtsi gave more calls of ‘present’ that were consistent with Bgee data, compared with tm1aWtsi. Whilst statistically significant, the effect size was small. The molecular basis of this observation was beyond the scope of the study, but the presence of selectable marker cassettes or short DNA sequences can interfere with the expression of neighbouring genes (Fiering et al., 1993; Hug et al., 1996; Olson et al., 1996; Pham et al., 1996; Meier et al., 2010; Maguire et al., 2014), and this may be true for the reporter gene element.

Interestingly, a pattern emerged in the data relating to homozygous viability. Genes causing lethality when inactivated were more likely to be expressed in a large number of tissues [Fig. 5 and Table S3; *P*<0.0001, Fisher's exact test comparing the ratio of viable and abnormal viability (subviable and lethal combined) gene sets between the groups with 0-3 and 31-39 positive structures]. No single structure dominated in this association with homozygous viability. This pattern is consistent with the increased lethality rates observed amongst the genes targeted with promoterless constructs in this data set, and reported previously (White et al., 2013). Genes causing lethality when inactivated were also more likely to be involved in a greater number of biological processes based on gene ontology annotations. As the data set grows, it may be possible to join the gene-expression and gene-ontology observations to build a predictor of viability.

Results presented herein demonstrate the potential of this resource to complement and extend existing knowledge and to stimulate and support hypothesis generation. For example, consistent with previous data indicating a role in the proximal convoluted tubules of the kidney (Vallon et al., 2011), *Slc5a2* was expressed in the cortex of the kidney, which is where these structures are located. Furthermore, supporting the hypothesis that *Ctnnal1* is a candidate gene for Hirschsprung disease (Tang et al., 2010a), here we show that the gene is expressed in the enteric nervous system, which is the primary site of disease manifestation.

As independent proof of the utility of expression data, this resource has been used previously to support single-gene, organ-specific and genome-wide studies. An example at the single-gene level is the characterisation of the role of keratin 76 in the normal function of tight junctions and maintenance of the skin barrier (DiTommaso et al., 2014a). Examples of application at the organ specific level include large-scale reverse genetics screens identifying genes required for normal skin function (DiTommaso et al., 2014b) and genetic determinants of bone mass and strength (Bassett et al., 2012). A similar, targeted application using microarray and *in situ* hybridisation data was reported for secreted and transmembrane proteins (Tang et al., 2010b).

The Respiratory Development and Disease Research Consortium, one of several Medical Research Council mouse networks established to focus resources and effort on key research areas, also recognised the utility of our expression data resource (Dean et al., 2013). In our current data set, 53% of genes presented with expression in the respiratory system (lung and/or trachea). Interestingly, the two organs in which reporter gene expression was most commonly observed were the brain and testis. This was the same trend reported by West et al. (2015), although the actual frequencies were different [brain ∼50%, male gonads ∼42% (West et al., 2015); brain 74.3%, testis 81.0% (present study)], which may reflect technical variations in the methodology resulting in different levels of sensitivity. The high percentage of genes expressed in the brain presumably reflects the heterogeneous nature of cell types and structures in the brain as well as the complexity of function and high levels of activity of those cells. The high percentage of genes expressed in the testis was reported previously (Schultz et al., 2003; Shima et al., 2004; Wu et al., 2004) and is considered reasonable given the unique functional nature of Sertoli, peritubular myoid and Leydig cells as well as the specialized requirements associated with meiosis and spermatogenesis and the requirements placed on sperm to facilitate fertilization (Shima et al., 2004). An interesting adjunct to this interpretation relates to fertility phenotyping. The homozygous infertility rate for this resource was reported previously as 5.2% (White et al., 2013). This was strongly biased towards gene knockouts specifically exhibiting male infertility (73.3%), compared with 6.7% exhibiting infertility restricted to females and 20% of genes in which infertility was observed in knockout mice of both sexes (White et al., 2013). Inactivation of *Sms* was reported by us previously to phenocopy many aspects of X-linked Snyder-Robinson syndrome (White et al., 2013). We also reported that males hemizygous for the null allele were infertile, a clinical feature that was not described in individuals with Snyder-Robinson syndrome. *Sms* was broadly expressed, detected in 25 entities, including all six of the sex-specific reproductive organs (Table S1).

In addition to the use of this type of data resource in single-gene and organ-specific studies of gene function and disease mechanisms, there is also precedent for genome-wide applications in an academic (White et al., 2013; West et al., 2015) and industrial context (Valenzuela et al., 2003; Moore, 2005). Complementing these data-generation efforts is the development of informatics tools to facilitate computational analysis of predicted tissue-phenotype associations for phenotypic observations in genetically altered mice (Oellrich et al., 2014). Taken together, these resources represent a rich source of information to extend the functional annotation of the mammalian genome and relate these observations to disease models and mechanisms.

## MATERIALS AND METHODS

### Ethics statement

The care and use of all mice in this study was in accordance with the UK's Animals in Science Regulation Unit's *Code of Practice for the Housing and Care of Animals Bred, Supplied or Used for Scientific Purposes* and the Animals (Scientific Procedures) Act 1986 Amendment Regulations 2012, and all procedures were carried out under a Home Office Project licence (no. 80/2485), which has been reviewed and approved by the Sanger Institute's Animal Welfare and Ethical Review Body.

### Animals

Mice were generated by blastocyst injection of targeted embryonic stem cells from the European Conditional Mouse Mutagenesis (EUCOMM) programme or the Knockout Mouse Project (KOMP; Skarnes et al., 2011). All mice used for this study contained either a *lacZ*-reporter-tagged knock-in allele, typically designated tm1a(EUCOMM)Wtsi or tm1a(KOMP)Wtsi, or a *lacZ*-reporter-tagged deletion allele, tm1b(EUCOMM)Wtsi or tm1b(KOMP)Wtsi, in which the critical exon had been removed by *Cre/loxP*-mediated excision (Fig. S1). For brevity, these alleles are referred to as tm1aWtsi and tm1bWtsi, respectively. The gene name and full allele symbol for each mutant mouse line in this study are presented in Table S1. Mice were maintained on an inbred C57BL/6N background (239 lines) or on mixed C57BL/6 backgrounds (210 lines composed of combinations of C57BL/6N, C57BL/6-*Tyr^c-Brd^*, C57BL/6NTac and C57BL/6Dnk; for details see Table S1, column BO). A total of 979 mutant mice were assessed from these 449 mouse lines. This encompassed 424 unique genes. For a subset of 25 randomly selected genes, the expression profile in both the knock-in allele and the deletion allele was assessed.

### Animal husbandry

Mice were maintained in a specific pathogen-free unit on a 12 h light:12 h dark cycle. The ambient temperature was 21±2°C and the humidity was 55±10%. Mice were given water and Mouse Breeders Diet (Lab Diets 5021-3 IPS, London, UK) *ad libitum* unless otherwise stated.

### Phenotypic characterisation

The homozygous viability of each mouse line was assessed as described previously (see Fig. S2A; White et al., 2013). Furthermore, mutant mice, along with age-, sex- and genetic-background-matched controls, were analysed using the standard Sanger MGP primary phenotyping screen as described previously (see Fig. S2B; White et al., 2013). All phenotyping results are available online (www.mousephenotype.org).

### Reporter gene detection

β-galactosidase (*lacZ*) reporter gene expression was assessed by whole-mount staining of up to 47 tissues collected from 6- to 30-week-old heterozygous or hemizygous mutant mice. Typically, two mice (one male and one female) were assessed per targeted gene, although this ranged from two to eight mice (Table S1). All mice were processed for whole-mount staining as described previously (Adams and Gale, 2006). Briefly, under terminal anaesthesia the mice were perfused with cold 4% paraformaldehyde (PFA, pH 8) before tissues were collected and fixed by immersion in 4% PFA for a further 30-60 min. After rinsing in PBS (pH 8), a slicer matrix (Agar Scientific, Stansted, UK) was used to cut thick (1 mm) midline sections of the brain (longitudinal) and kidney (transverse), and a transverse bisection of the spleen was performed. Tissues and sections were incubated in staining solution containing 0.1% X-Gal (5-bromo-4-chloro-3-indolyl-β-d-galactoside; Invitrogen, Paisley, UK) at 4°C for 48 h followed by a post-fixation with 4% PFA overnight at 4°C. Clearing of the tissues was performed in glycerol, and the resulting resource was stored in the dark at room temperature. Reporter gene expression was manually assessed and recorded in all tissues and, where staining was observed, standardised images were collected using a Leica microscope and LAS v4 software. The location of stain on images was annotated with Mouse Anatomy (MA) ontology terms. All expression data are available online (www.mousephenotype.org). Furthermore, the resulting samples were stored as part of a biobank of biological resources that can be accessed upon request. The longevity of this resource was assessed over a period of 2 years. Whilst much staining was preserved after 2 years, there was a yellow discoloration of the samples with time and some diffusion of stain occurred, particularly from adipose tissue. For optimal results, annotation and imaging was performed within 1 month of staining.

The data collected on individual mice were used to derive summary expression profiles for each gene, for up to 47 tissues assessed (Table S1) using the following strategy and associated rules. (1) The most confident calls were derived from multiple mice and were fully concordant. (2) For the majority of reproductive tissues, and occasionally when other tissues were missing, calls were accepted from only one animal. (3) For the remainder, where the calls were combined from more than one animal, calls of ‘present’ and ‘not detected’ were made only if the results from at least half the mice were in agreement. (4) ‘Present’ calls were given precedence, therefore ‘not detected’ calls were made only if half or more mice did not detect staining in the structures and there were no ‘present’ calls. (5) ‘Ambiguous’ calls were made if there was <50% agreement to justify the ‘present’ or ‘not detected’ calls, or if only ‘ambiguous’ calls were made. (6) ‘No data’ calls were discarded so that they had no influence when calls were combined and, for some structures, no result was given.

To assess the occurrence of endogenous background, adult wild-type mice on C57BL/6N and on mixed C57BL/6 backgrounds (combinations of C57BL/6N, C57BL/6-*Tyr^c-Brd^*, C57BL/6NTac and C57BL/6Dnk) were stained, examined and imaged. Furthermore, to monitor the stability of the background interference, one male and one female wild-type control was included each time mutant mice were processed. A total of 116 wild-type adult mice were screened (60 females and 56 males).

### Bioinformatic analysis

GO term enrichment was assessed with FuncAssociate v2.0 (Berriz et al., 2009; http://llama.mshri.on.ca/funcassociate/) using a gene association file downloaded from ftp://ftp.geneontology.org/ on 23 June 2014. FuncAssociate was configured to exclude computationally predicted GO annotations (IEA evidence code). Revigo reduced the GO term redundancy by clustering significant terms into representative subsets (Supek et al., 2011). Comparison with published adult mouse (>6 weeks old) expression data was performed using the Bgee gene expression database [Bastian et al., 2008; release version 12; http://bgee.unil.ch/bgee/bgee; stage MmusDO:0000040; customised, mouse-specific data release (personal communication Frédéric Bastian, University of Lausanne, Switzerland)].
